# Silk peptide treatment potentiates natural killer cell activity *in vitro* and induces natural killer cell maturation and activation in mouse splenocytes

**DOI:** 10.1080/13880209.2019.1617749

**Published:** 2019-06-02

**Authors:** Sun-Hee Jang, Mi-Sun Oh, Hyang-Im Baek, Ki-Chan Ha, Jeong-Yong Lee, Yong-Suk Jang

**Affiliations:** a Department of Molecular Biology and the Institute for Molecular Biology and Genetics, Chonbuk National University, Jeonju, Korea;; b Worldway Co., Ltd, Sejong, Korea;; c Healthcare Claims and Management Inc, Jeonju, Korea;; d Department of Bioactive Material Sciences and Research Center of Bioactive Materials, Chonbuk National University, Jeonju, Korea

**Keywords:** Cytolytic activity, complementary therapy, *ex vivo*, target cell lysis

## Abstract

**Context:** Silk peptide from cocoons of silkworm (*Bombyx mori* L., Bombycidae) has been employed as a biomedical material and exhibits various bioactivities, including immune-modulating activity.

**Objective:** We analyzed whether silk peptide exerts direct modulating effects on NK cells using an NK cell line *in vitro* and *ex vivo* splenocytes. We also attempted to delineate the mechanism underlying the modulation.

**Material and methods:***In vitro* activity of silk peptide on NK cells was determined by measurement of cytolytic activity against K562 cells at an effector-to-target ratio of 5:1 after incubation of NK-92MI cells with silk peptide (0–2000 μg/mL) for 48 and 72 h. *Ex vivo* activity of silk peptide on mouse splenic NK cells was determined similarly by using YAC-1 cells.

**Results:** Treatment of NK-92MI NK cells with silk peptide (500–2000 μg/mL) significantly increased cytolytic activity on target cells by 2- to 4-fold. The same concentrations (500–2000 μg/mL) of silk peptide treatment also significantly enhanced the cytolytic activity of splenic NK cells against YAC-1 cells. Silk peptide treatment of IL-2-stimulated splenocytes induced enhanced expression of Th1, 2 and 17 cytokines including TNF-α, IFN-γ, IL-6, IL-4 and IL-17. Finally, *ex vivo* treatment with silk peptide on mouse splenocytes significantly enhanced the degree of NK cell maturation in a dose-dependent manner from 3.49 to 23.79%.

**Discussion and conclusions:** These findings suggest that silk peptide stimulates NK cells, thereby influencing systemic immune functions and improving natural immunity. Thus, silk peptide could be useful as a complementary therapy in cancer patients.

## Introduction

Cancer, one of the most lethal and intractable diseases, is commonly treated by surgery, chemotherapy and irradiation. However, serious side effects of these treatments, including normal cell cytotoxicity, metastasis and recurrence of cancer, remain serious obstacles to effective treatment. The main focus of current research on cancer therapy is to minimize the cancer mass using existing treatment methods, followed by removal of residual cancer cells through application of immunotherapeutic strategies. In particular, cell-based immunotherapies depend on the immune system removing cancer cells by activating the cells involved in immune surveillance within the body (Guedan et al. 2019). Since the activity of these immune cells is seriously impaired in cancer patients, therapeutic agents for cancer that potentiate immune cells have been actively developed to strengthen the function of immune cells that selectively destroy cancer cells (Pejin et al. 2014; Eyileten et al. 2016; Pejin and Glumac 2018). Among these cell types, natural killer (NK) cells are attractive candidates for cell-based immunotherapy because they are effective in directly preventing the development, proliferation, metastasis and recurrence of cancer cells (Kärre et al. 1986), and have been shown to play a key role in the removal of cancer and virus-infected cells (Vivier et al. 2008). NK cells are typically dormant, only infiltrating tissues when activated (Glas et al. 2000). Several soluble mediators, including cytokines, chemokines and soluble receptor ligands, as well as cell-to-cell interactions, are involved in the regulation of NK cell functions (Smyth et al. 2002). Among the cytokines, type I interferon (IFN), interleukin (IL)-12 and IL-18 are potent activators of NK cell effector function (Walzer et al. 2005). Upon activation by various stimuli, including surface receptors such as natural cytotoxicity receptors and NK group 2, member D (NKG2D), NK cells release cytotoxic granules and secrete cytokines such as IFN-γ to induce death of target cells (Vivier et al. 2008).

Since the 1970s, many bioactive peptides derived from food protein hydrolysates have been studied as potential nutraceuticals (Nagai et al. 2009; Kim et al. 2011). Dietary proteins and amino acids are important for immune function, and some act as immune response modulators (Li et al. 2007; Guadagni and Biolo 2009). Among these dietary proteins, silk peptide has been proposed as a bioactive supplement (Teramoto et al. 2008). Silk peptide, which is considered a healthy functional food in Asian countries, comprises natural biomolecules that are also used in powder and extract forms for pharmacological applications, as well as in the biomedical and biotechnological fields (Lee et al. 2012). Silk peptide biopolymers, which are similar to proteins such as collagen, elastin, keratin, fibroin and spongin, are produced by silkworms to form cocoons for protection from the environment during metamorphosis to the mature moth stage (Ahmad et al. 2004; Seo et al. 2011). Silk peptide displays diverse bioactivities, such as anti-inflammatory, immune-regulatory, antitumor, antiviral and antibacterial properties (Singh et al. 1990; Gotoh et al. 2000; Jung et al. 2010). Importantly, silk fibroin peptides and silk protein sericin have been reported to inhibit the proliferation of human lung cancer cells, induce apoptosis, inhibit the formation of colorectal tumors and exert anticancer effects against cancer cells (Zhaorigetu et al. 2001; Kaewkorn et al. 2012; Wang et al. 2019).

Previous studies have demonstrated that silk peptide activates macrophages, enhancing their phagocytic ability and stimulate the production of pro-inflammatory cytokines (Uff et al. 1995). Silk peptide also induces strong tumor necrosis factor production, and weaker production of IL-1β and IL-6 in RAW 264.7 cells (Cui et al. 2013). However, studies on the effects of silk peptide on NK cell activity, a promising candidate for cell-based cancer immunotherapy, are scarce. In a previous study, we reported that oral administration of acid-hydrolyzed silk peptide to mice exerted immune-modulating activity (Jang et al. 2018). In particular, oral silk peptide treatment significantly enhanced the frequency and degree of maturation of NK cells in splenocytes of treated mice. This treatment also significantly enhanced the cytolytic activity of NK cells in the splenocytes of treated mice. In this study, we investigated whether silk peptide exerts direct modulating activity on NK cells using an NK cell line *in vitro* and splenic NK cells *ex vivo*.

## Materials and methods

### Preparation of acid-hydrolyzed silk peptide

The silk peptide used in this study was prepared from cocoons of silkworm, which had been identified and distributed by Rural Development Administration of Korea. The silk peptide is an ingredient used by Worldway Co., Ltd. (Sejong, Korea) to manufacture commercial products. Briefly, silk worm cocoons were hydrolyzed, followed by neutralization, decolorization, filtration, desalting, concentration and drying to obtain a pale yellow powder. Silk peptide (5 mg) was dissolved in 20 mM HCl solution (100 mL) and derivatized. Derivatized samples were quantitated using a Waters 2695 Alliance high-performance liquid chromatography (HPLC) system (Waters Co., Milford, MA) and a Waters 2475 Multi-λ fluorescence detector. The amino acid composition of the silk peptide used in this study was as follows: alanine, 20.0%; glycine, 31.3%; serine, 15.3% and other components, 17.95%.

### Experimental materials

Unless otherwise specified, the chemicals and laboratory supplies used in this study were purchased from Sigma Chemical Co. (St. Louis, MO, USA) and SPL Life Sciences (Pocheon, Korea), respectively. Fetal bovine serum (FBS), RPMI-1640, and α-minimal essential medium (α-MEM) media were obtained from Hyclone Laboratories (Logan, UT, USA). The mouse lymphoma YAC-1 cell line was kindly provided by C. Kim (Inha University, Incheon, Korea). NK-92MI NK cells and their target K562 cells (a human erythroleukemia cell line) were kindly provided by Dr. D. Cho (Korea University, Seoul, Korea). The Cytometric Bead Array Mouse Th1/Th2/Th17 Cytokine Kit for cytokine detection was purchased from BD Pharmingen (Franklin Lakes, NJ, USA). ^3^H-TdR was purchased from Perkin-Elmer Life and Analytical Sciences (Shelton, CT, USA).

### Antibodies (abs) and flow cytometric analyses

The following Abs against mouse molecules were purchased from Miltenyi Biotec Inc. (Bergisch Gladbach, Germany): CD3-fluorescein isothiocyanate (FITC), NK1.1-peridine-chlorophyll-protein (PerCP)-Vio700, CD11b-allophycocyanin (APC)-Vio770, CD27-phycoerythrin (PE)-Vio770, NKG2D-APC, NKp46-PE-Vio770, KLRG1-PE, Ly49D-PE, CD4-PerCP-Vio700 and CD8a-APC for mouse molecules, and CD56-FITC, CD107a-PE and IFN-γ-APC for human molecules. We purchased 7-amino-actinomycin D (7-ADD) and FITC-conjugated annexin V from BD Biosciences (San Jose, CA, USA). Flow cytometric analysis was performed using a Cytoflex flow cytometer (Beckman Coulter, Inc., Brea, CA, USA) and data analyses were performed using the CytExpert software (Beckman Coulter, Inc.).

### Measurement of silk peptide-mediated cytotoxic and mitogenic effects on NK-92MI NK cells

Possible silk peptide-mediated cytotoxicity against NK-92MI NK cells was assessed using the 3-(4,5-dimethylthiazol-2-yl)-2,5-diphenyltetrazolium bromide (MTT) method after incubating NK-92MI cells (1 × 10^5^) with various concentrations (0, 100, 500, 1000 and 2000 μg/mL) of silk peptide. Briefly, after incubating the cells with silk peptide for 48 or 72 h at 37 °C in a CO_2_ incubator, 50 µL serum-free media and 50 µL MTT solution (1 mg/mL) were added into each well and further incubated for 4 h. Next, 50 µL dimethyl sulfoxide (DMSO) was added into each well and incubated for 15 min at 37 °C. Color development was measured by reading the absorbance at 540 nm on an enzyme-linked immunosorbent assay (ELISA) plate reader (SPECTROstar Nano, BMG Labtech, Ortenberg, Germany).

Silk peptide-mediated NK-92MI cell proliferation was determined using a ^3 ^H-TdR incorporation assay. Briefly, NK-92MI cells (1 × 10^5^) were treated with various concentrations (0, 100, 500, 1000 and 2000 μg/mL) of silk peptide for 48 or 72 h in a CO_2_ incubator at 37 °C, and then 0.5 μCi ^3 ^H-TdR was added to each well. After a 12-h chase incubation, the cells were collected using a 96-well cell harvester (Inotech, Dottikon, Switzerland), and the incorporated tritium content was quantified using a 1450 Microbeta liquid scintillation counter (Perkin-Elmer Life and Analytical Sciences). Stimulation indices were calculated by dividing the tritium incorporation (cpm) in cells treated with the sample with that in control cells treated with culture medium (α-MEM with 15% FBS).

### Silk peptide-mediated NK-92MI cell activation and target cell cytotoxicity assay

To measure NK cell activation, the cell surface expression of CD107a, a marker for NK cell degranulation, and intracellular IFN-γ expression, a marker for NK cell activation, were determined (Betts et al. 2003; Aktas et al. 2009). Briefly, to determine degranulation after NK cell activation, NK-92MI NK cells (1 × 10^6^ cells/mL) were stimulated with various concentrations of silk peptide for 72 h and then incubated with K562 target cells at an effector-to-target ratio of 5:1, or without target cells in the presence of brefeldin A (10 μg/mL) and monensin (6 μg/mL) for 6 h at 37 °C in a CO_2_ incubator. The level of NK cell degranulation was measured via flow cytometry after adding FITC-conjugated anti-CD56 Ab and PE-conjugated anti-CD107a Ab. Similarly, intracellular IFN-γ expression was determined through flow cytometry using APC-conjugated anti-IFN-γ Ab.

Silk peptide-treated NK-92MI NK cell-mediated target cell cytotoxicity was measured as described previously (Aubry et al. 1999; Jang et al. 2018). Briefly, K562 target cells (2 × 10^7^ cells/mL) were labeled with Paul Karl Horan (PKH)-26 and mixed with silk peptide-stimulated NK-92MI effector cells at various effector-to-target ratios. Cells were plated and incubated for 4 h at 37 °C in a CO_2_ incubator. Following incubation, 7-ADD and FITC-annexin V were added to determine the level of apoptosis induced in K562 target cells via flow cytometry.

### Determination of NK cell activity using *ex vivo* splenocytes

We prepared splenocytes from mice and the degree of NK cell maturation was determined through flow cytometry by measuring the expression levels of CD3, NK1.1, CD27 and CD11b. To measure NK cell-mediated cytotoxicity, YAC-1 target cell lysis was measured as described previously (Aubry et al. 1999). Briefly, YAC-1 target cells were labeled with PKH-26 and mixed with effector cells at an effector-to-target ratio of 20:1. Cells were plated and incubated for 4 h at 37 °C in a CO_2_ incubator. Following incubation, 7-ADD and FITC-annexin V were added to determine the level of apoptosis induced in target cells via flow cytometry.

### Analysis of cytokine expression in *ex vivo* splenocytes

To analyze cytokine production, splenocytes (2 × 10^5^) were distributed into each well of a 24-well plate and stimulated with phorbol 12-myristate 13-acetate (PMA, 50 ng/mL) and ionomycin (0.5 μg/mL) for 4 h. The culture medium was collected after stimulation and expression levels of various cytokines were measured using a CBA mouse Th1/Th2/Th17 cytokine kit (BD Pharmingen) according to the manufacturer’s recommendations. The results were analyzed using FCAP Array software (BD Biosciences). Cytokine concentrations were calculated using a standard curve generated from cytokine standards.

### Determination of CD4/CD8 ratio, NK cell maturation and NK cell activation receptor expression in *ex vivo* splenocytes

Following the incubation of splenocytes (1 × 10^6^ cell/mL) with various concentrations of silk peptide (0, 50, 100, 200 and 500 μg/mL), the CD4^+^/CD8^+^ cell ratio was determined from CD3^+^ cells via flow cytometry. The degree of NK cell maturation was then determined through flow cytometry according to the expression levels of CD3, NK1.1, CD27 and CD11b (Hayakawa et al. 2006; Chiossone et al. 2009). Finally, the cell surface expression levels of NK cell activation receptors was determined through flow cytometry according to the expression levels of NKG2, NKp46, KLRG1 and Ly49D in CD3^+^NK1.1^+^ cells.

### Statistical analyses

All data are presented as means ± standard deviation (SD) and all experiments were repeated at least three times. All statistical analyses were performed using GraphPad Prism (GraphPad Software Inc., La Jolla, CA, USA) and SigmaPlot software (ver. 12.0; Systat Software, San Jose, CA, USA). Statistical significance was determined using unpaired two-tailed Student's *t*-tests at a significance level of *p* < 0.05.

## Results

### Silk peptide did not show prominent cytotoxic or mitogenic activity on NK-92MI NK cells

**Figure 1.**
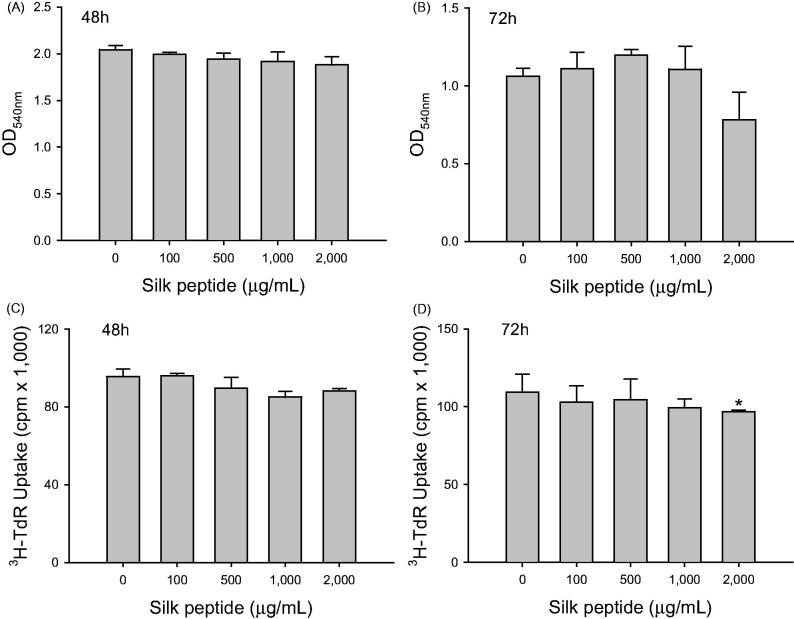
Direct cytotoxic and mitogenic activity of silk peptide in NK-92MI NK cells. NK-92MI cells were treated with various concentrations of silk peptide *in vitro*. Cytotoxicity in NK-92MI cells was measured at (A) 48 h and (B) 72 h after incubation at indicated silk peptide concentrations. NK-92MI cell proliferation was measured at (C) 48 h and (D) 72 h after treatment with the indicated silk peptide concentrations. Data are presented as means ± standard deviation (SD) of triplicate measurements. A representative result of at least three independent experiments is shown. Asterisks (*) indicate significant differences compared with control (*p* < 0.05).

For preliminarily analysis of the direct effects of silk peptide on NK cell activation, which was observed in earlier *in vivo* experiments (Jang et al. 2018), we measured the cytotoxic and mitogenic activity of silk peptide at various concentrations on NK-92MI NK cells (Figure 1). We detected no statistically significant cytotoxic or mitogenic activity of silk peptide on NK cells after 48 h of treatment (Figure 1(A,C)). We also detected no statistically significant cytotoxicity of silk peptide at any of the concentrations tested after 72 h of treatment (Figure 1(B)). However, we observed significant inhibition of cell proliferation when the highest silk peptide concentration (2 mg/mL) was applied for 72 h (*p* < 0.05), although significant inhibition of NK cell proliferation was not detected at the other concentrations (Figure 1(D)). Collectively, these results suggest that silk peptide has no substantial cytotoxic or mitogenic activity in NK-92MI NK cells, and that NK cell activation might be achieved via activation of signaling of NK cells rather than an increase in cell numbers.

### *In vitro* treatment of NK-92MI NK cells with silk peptide enhances cytolytic and functional activity of the NK cells

**Figure 2.**
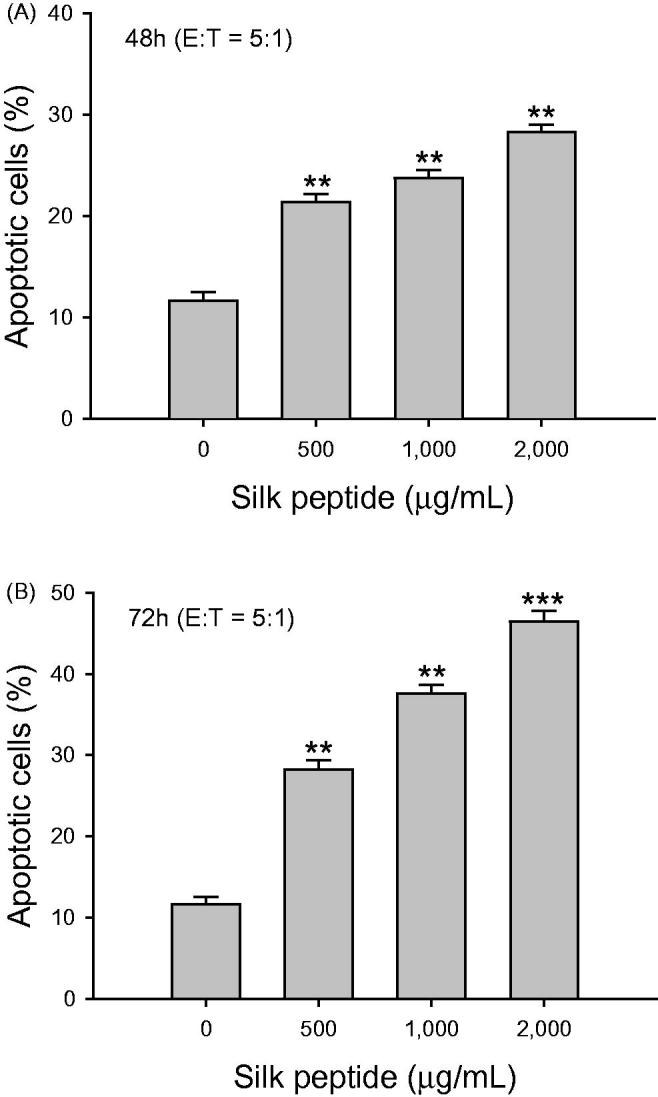
Cytolytic activity of *in vitro* silk peptide-treated NK-92MI cells on K562 target cells. NK-92MI cells were treated with the indicated silk peptide concentrations for (A) 48 h and (B) 72 h, and incubated with PKH-26-labeled YAC-1 target cells at an effector-to-target ratio of 5:1. The degree of target cell lysis was measured as described in the Materials and methods. Data are presented as means ± SD of triplicates. A representative result of at least three independent experiments is shown. E:T, effector-to-target ratio. Asterisks (*) indicate significant differences compared with control (**p < 0.01, ***p < 0.001).

**Figure 3.**
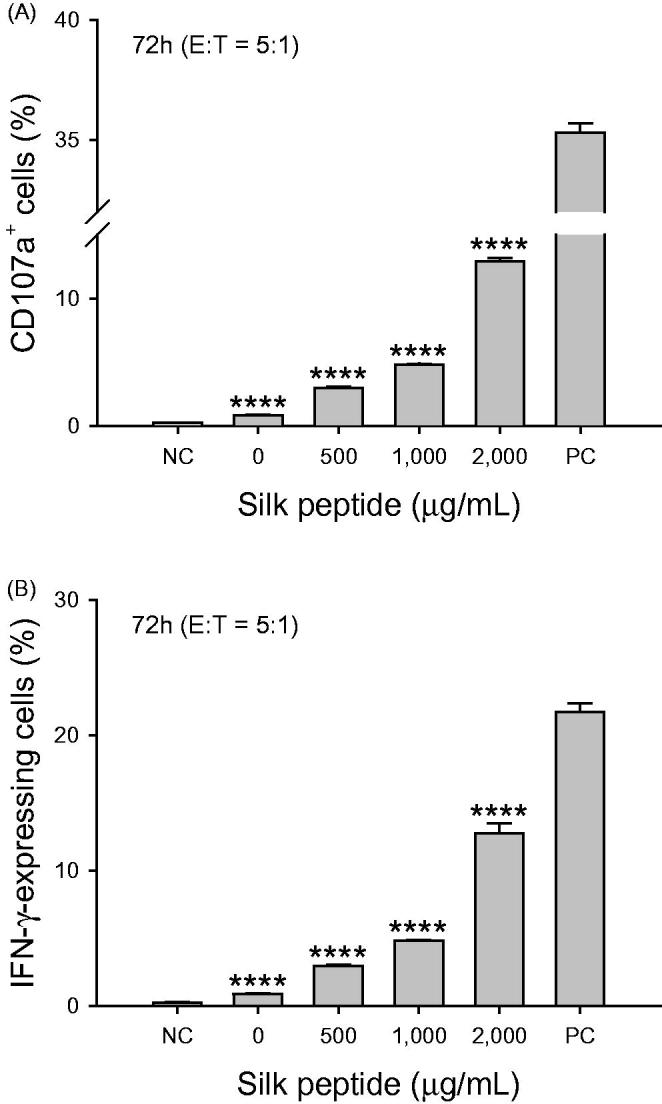
Level of NK cell activation determined by cell surface CD107a expression and intracellular interferon (IFN)-γ expression in NK-92MI cells after silk peptide treatment *in vitro*. NK-92MI cells were treated with the indicated silk peptide concentrations for 72 h and incubated with K562 target cells at an effector-to-target ratio of 5:1 in the presence of brefeldin A and monensin. (A) Cell surface CD107a expression and (B) intracellular IFN-γ expression were assessed via flow cytometry. Data are presented as means ± SD of triplicates. A representative result of at least three independent experiments is shown. Asterisks (*) indicate significant differences compared with control (****p < 0.0001). NC and PC represent negative control (unstimulated control containing NK cells without target cells) and positive control (NK cells stimulated with PMA and ionomycin), which indicate minimum and maximum levels, respectively.

We next explored the direct enhancement by silk peptide treatment of NK-92MI NK cell activity (Figure 2). On treating NK-92MI cells with various concentrations of silk peptide for 48 and 72 h and incubating them with K562 cells at an effector-to-target cell ratio of 5:1, the cytolytic activity of NK-92MI cells increased significantly in a dose-dependent manner by about 2- to 4-fold (*p* < 0.01 and *p* < 0.001). We then examined markers for functional NK cell activation, cell surface CD107a expression and intracellular IFN-γ expression, in NK-92MI cells after treatment with various concentrations of silk peptide for 72 h and incubation with K562 target cells at an effector-to-target cell ratio of 5:1 (Figure 3). We determined that the frequency of cells expressing CD107a, a functional marker for the excretion of cytolytic granules by NK cells, increased significantly following *in vitro* silk peptide treatment in a dose-dependent manner (*p* < 0.0001; Figure 3(A)). The frequency of cells expressing intracellular IFN-γ, the most critical cytokine for NK cell activation, also increased significantly in a dose-dependent manner following *in vitro* silk peptide treatment (*p* < 0.0001; Figure 3(B)).

### Silk peptide treatment of *ex vivo* mouse splenocytes enhanced target cell cytolytic activity

**Figure 4.**
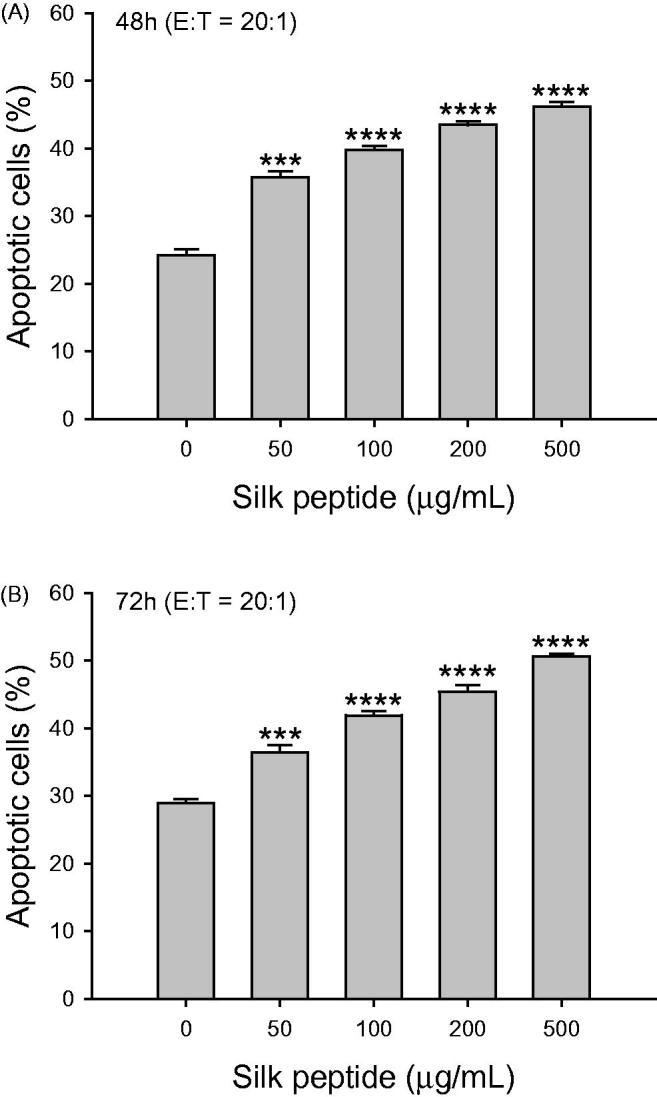
Cytolytic activity of *ex vivo* silk peptide-treated mouse splenocytes on YAC-1 target cells. Splenocytes were treated with the indicated silk peptide concentrations for (A) 48 h and (B) 72 h, and incubated with PKH-26-labeled YAC-1 target cells at an effector-to-target ratio of 20:1. The degree of target cell lysis was measured as described in the Materials and methods. Data are presented as means ± SD of triplicates. A representative result of at least three independent experiments is shown. Asterisks (*) indicate significant differences compared with control (***p < 0.001, ****p < 0.0001).

We then attempted to confirm that the enhancement of NK cell activation by silk peptide in the NK-92MI NK cell line could also be achieved in splenic NK cells. Initially, we tested whether silk peptide treatment itself had cytotoxic or mitogenic effects on mouse splenocytes, and did not detect any such effects at concentrations <1 mg/mL (data not shown). We treated mouse splenocytes with various concentrations (0–500 μg/mL) of silk peptide for 48 and 72 h and incubated then with YAC-1 cells at an effector-to-target cell ratio of 20:1. We found that the cytolytic activity of silk peptide-stimulated splenocytes increased significantly in a dose-dependent manner (*p* < 0.001 and *p* < 0.0001). We therefore conclude that silk peptide treatment of mouse splenocytes induced enhanced cytolytic activity against YAC-1 cells (Figure 4).

### Silk peptide treatment of *ex vivo* mouse splenocytes stimulated expression of critical cytokines for immune regulation

**Figure 5.**
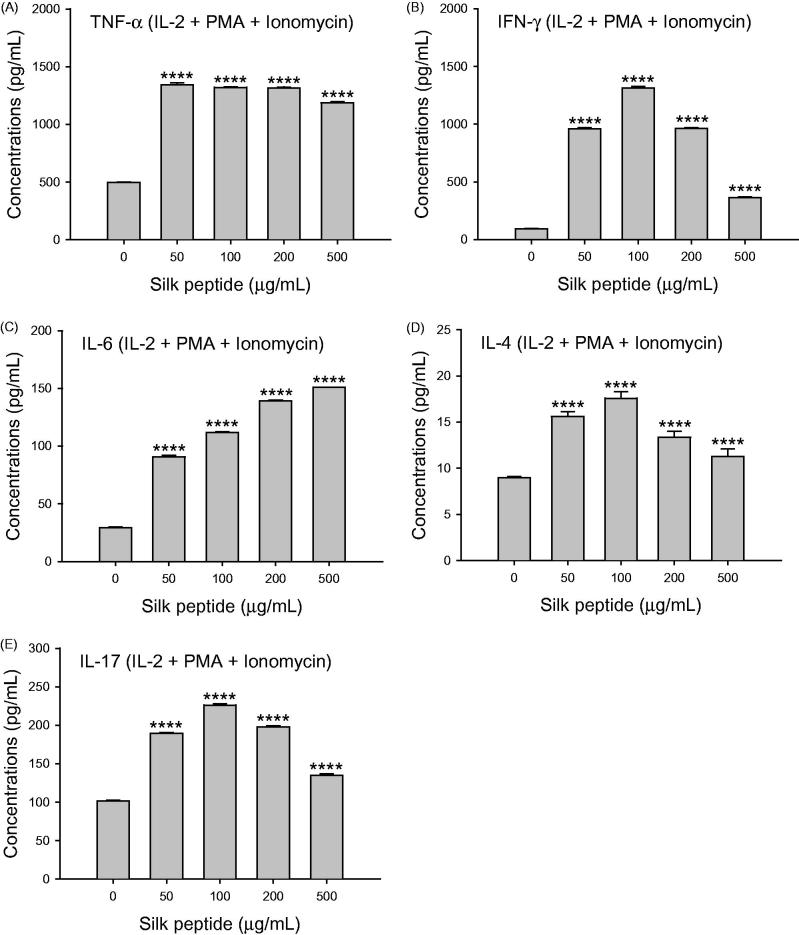
Cytokine expression levels following treatment of splenocytes with the indicated silk peptide concentrations and IL-2, PMA, and ionomycin. Expression levels of the Th1-type cytokines (A) tumor necrosis factor (TNF)-α and (B) IFN-γ; the Th2-type cytokines (C) interleukin (IL)-6 and (D) IL-4; and (E) the Th17-type cytokine IL-17 were determined as described in the Materials and methods. Data are presented as means ± SD of triplicates. A representative result of at least three independent experiments is shown. Asterisks (*) indicate significant differences compared with control (****p < 0.0001).

Given that silk peptide exerted no mitogenic effects on the NK cell line (Figure 1) or mouse splenocytes (data not shown), silk peptide may be involved in the activation of immune cell signaling. Therefore, we determined the expression levels of cytokines that are critically involved in immune stimulation (Figure 5). The expression of Th1-type cytokines (tumor necrosis factor (TNF)-α and INF-γ) increased significantly in the presence of IL-2, PMA, and ionomycin (*p* < 0.0001) (Figure 5(A,B)). Similarly, the expression of Th2-type cytokines (IL-4 and IL-6) also increased significantly (*p* < 0.0001; Figure 5(C,D)). This statistically significant increase in cytokine expression in silk peptide-stimulated splenocytes was also observed in the Th17-type cytokine, IL-17 (Figure 5(E)). Collectively, these results suggest that silk peptide functions as a stimulator of cellular immunity (Th1-type response), humoral immunity (Th2-type response), and adaptive immunity against pathogen infection (Th17-type response).

### Silk peptide treatment of *ex vivo* mouse splenocytes affected T cell subpopulation size and NK cell maturation

To characterize the effect of silk peptide on the activation of splenic lymphocytes, we next determined whether silk peptide treatment affects T cell subpopulation size in splenic lymphocytes. First, we found that silk peptide treatment significantly reduced the CD4/CD8 cell ratio in splenocytes compared with the control (1.36 ± 0.01 and 1.9 ± 0.01, respectively, *p* < 0.0001) (Figure 6(A)). We next examined changes in NK cell maturation following silk peptide treatment of mouse splenocytes (Figure 6(B)). Mouse NK cells were divided into four subsets based on the expression levels of CD27 and CD11b on their surface, as follows (naïve to mature phenotype): CD11b^low^CD27^low^, CD11b^low^CD27^high^, CD11b^high^CD27^high^, and CD11b^high^CD27^low^ (Hayakawa et al. 2006; Chiossone et al. 2009). The proportion of mature NK cells (23.79 ± 0.69%) increased significantly in a dose-dependent manner following silk peptide treatment (*p* < 0.0001). Therefore, silk peptide treatment may induce lymphocyte activation and NK cell maturation.

Figure 6.Influence of silk peptide treatment on T cell frequency and NK cell maturation in *ex vivo* mouse splenocytes. Changes in (A) the CD4/CD8 ratio and (B) the frequency of mature NK cells (CD11b^high^CD27^low^) in silk peptide-treated splenocytes were analyzed as described in the Materials and methods. The fluorescence-activated cell scan (FACS) plot shows a representative result. Data shown in the bar graph are means ± SD of triplicate results. A representative result of at least three independent experiments is shown. FSC-A, forward-scatter area; SSC-A, side-scatter area. Asterisks (*) indicate significant differences compared with control (****p < 0.0001).

**Figure:**
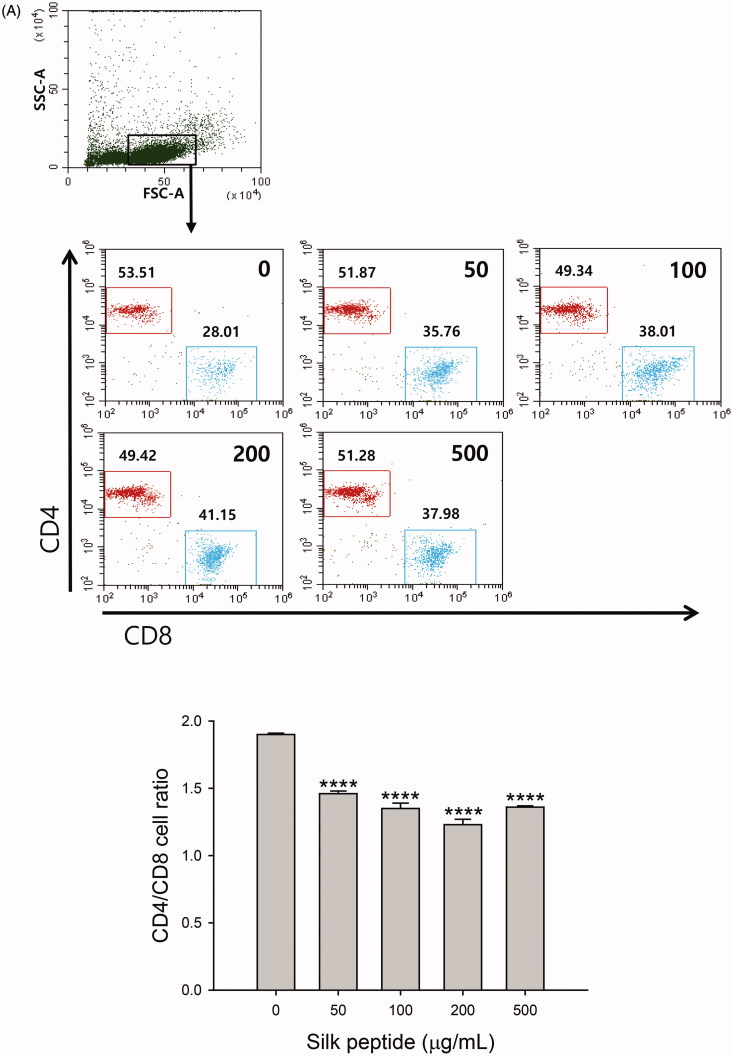

**Figure:**
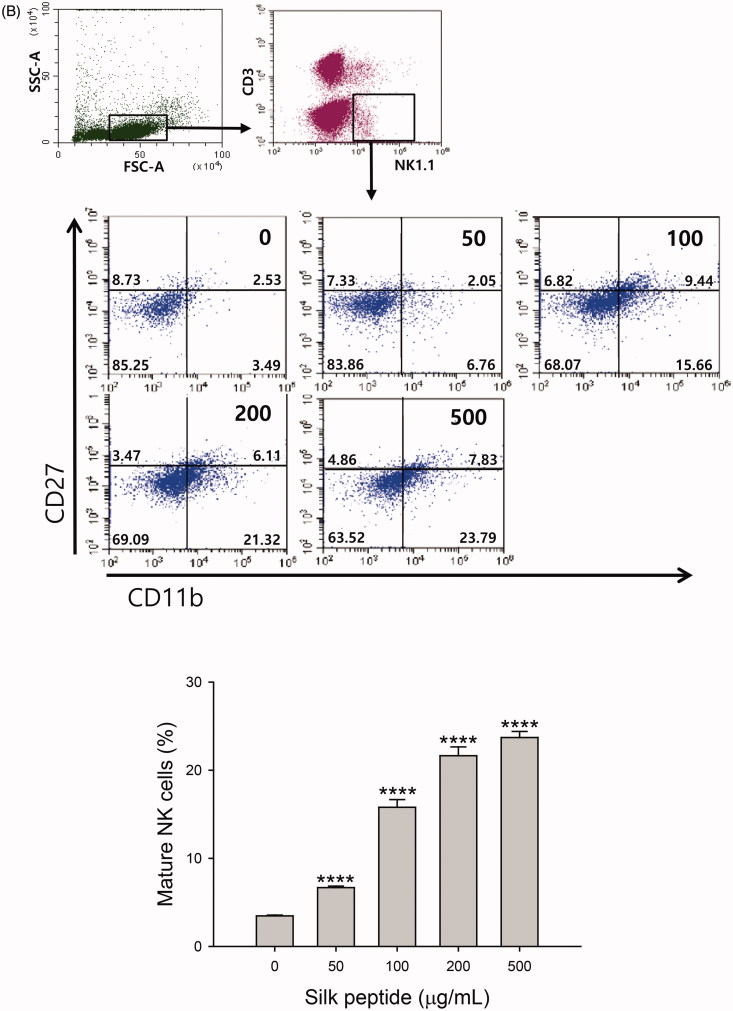

### Silk peptide treatment increased the expression of NK cell activation receptors

**Figure 7.**
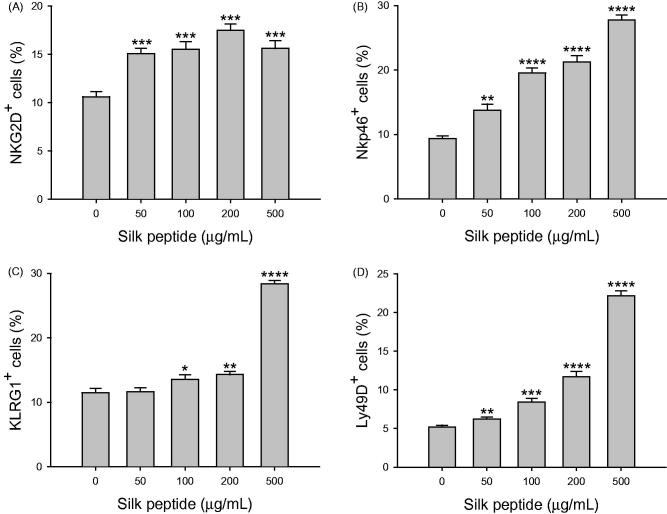
Effects of silk peptide treatment on the expression of NK cell-activating receptors in splenocytes. Splenocytes were treated with the indicated silk peptide concentrations for 48 h and expression levels of (A) NKG2D, (B) Nkp46, (C) KLRG1, and (D) Ly49D were analyzed as described in the Materials and methods. Data are presented as means ± SD of triplicate results. A representative result of least three independent experiments is shown. Asterisks (*) indicate significant differences compared with control (*p < 0.05, **p < 0.01, ***p < 0.001 and ****p < 0.0001).

The lack of any mitogenic effect and induction of maturation of NK cells following silk peptide treatment in mouse splenocytes suggests that silk peptide affects the activation of NK cells. Therefore, we determined whether silk peptide treatment in mouse splenocytes increases the expression of NK cell-activation receptors including NKG2D, NKp46, Ly49D and KLRG1 in CD3^+^NK1.1^+^ cells (Figure 7). As expected, and consistent with our findings on silk peptide-mediated enhancement of NK cell maturation (Figure 6(B)), silk peptide treatment in mouse splenocytes significantly enhanced the expression of cell surface receptors representing NK cells activation, including NKG2D, NKp46, KLRG1 and Ly49D. Collectively, these results suggest that silk peptide directly stimulates splenic NK cells.

## Discussion

Proper immune regulation is important for disease prevention and health throughout the lifespan in humans. Numerous studies have reported that decreased immune function weakens resistance to illness, while healthy food consumption strengthens the immune system. In particular, it has been reported that certain protein food products, including lactoferrin and soybean protein fraction (a milk-derived protein casein), possess immune-modulating functions that increase NK cell activity in immunocompromised mice, stimulate the cellular immune response and exert mitogenic effects (Kuhara et al. 2006; Egusa and Otani 2009; Chang et al. 2012). Silk protein has also been proposed to be a bioactive substance, and has shown efficacy in reducing blood pressure and treating atopic dermatitis, as well as exhibiting antitumor activity, promoting insulin release, and enhancing innate immune responses (Igarashi et al. 2006; Teramoto et al. 2008; Jung et al. 2010; Moon et al. 2011; Ikegawa et al. 2012). More importantly, silk fibroin peptides and silk protein sericin have been shown to inhibit human lung cancer cell proliferation, induce apoptosis, inhibit colorectal tumor formation and exert anticancer effects (Zhaorigetu et al. 2001; Kaewkorn et al. 2012; Wang et al. 2019).

Cytolytic activity in the immune system is essential for maintaining health, as it ensures the removal of pathogen-infected and malignantly transformed cells. NK cells are specialized cytotoxic innate immune cells that directly kill both virus-infected and abnormal cells (Topham and Hewitt 2009). In this context, we previously explored the effects of silk peptide on immune capability *in vivo* (Jang et al. 2018), specifically by promoting NK cell activity in mice. In this study, we investigated whether silk peptide modulates NK cells using NK cell line and *ex vivo* splenocytes. We observed increased NK-92MI NK cell activity (Figure 2) and increased expression of cell surface CD107a and intracellular IFN-γ (Figure 3) following treatment with silk peptide *in vitro*. Silk peptide-mediated enhancement of cytolytic activity against YAC-1 cells (Figure 4) and NK cell maturation (Figure 6) were also observed *ex vivo* in splenocytes. NK cells do not have antigen-specific receptors, instead expressing various germ-line encoded immune receptors that modulate NK cell activity. The ability of NK cells to recognize target cells is mediated by signaling based on the activation of NKG2D, Ly49D, DNAM-1, and natural cytotoxicity receptors. The fine balance and integration of signals from activating and inhibitory receptors regulates cytolytic activity in NK cells (Watzl and Long 2010). Importantly, we found that silk peptide treatment of *ex vivo* splenocytes increased the expression of NK cell activation markers including NKG2D, Nkp46, KLRG1 and Ly49D (Figure 7). These results suggest that silk peptide can induce NK cell activation signals that promote enhanced NK cell maturation and cytolytic activity.

The production of Th1-type cytokines is mainly activated by innate immune mechanisms that stimulate cellular immune responses, while Th2-type cytokines act as mediators of adaptive humoral immunity (Khan 2008). We observed that balanced expression of Th1- and Th2-type cytokine was induced by silk peptide treatment of splenocytes primed with IL-2 (Figure 5(A–D)). We also detected enhanced expression of Th17-type cytokine following silk peptide treatment of mouse splenocytes, which is known to promote the expression of chemokines recruiting immune cells, such as monocytes and neutrophils (Figure 5(E)). Interestingly, we also found that the CD4/CD8 ratio decreased in silk peptide-treated splenocytes (Figure 4); we detected no significant cytotoxic effect of silk peptide treatment, suggesting a preferential increase in CD8^+^ T cells rather than a decrease in CD4^+^ T cells (Figure 5) because we did not detect any significant cytotoxic effect of silk peptide treatment (data not shown). Given that CD8^+^ T cells are involved in antigen-specific cytotoxic immunity, silk peptide might also promote cytolytic immune function following silk peptide treatment.

## Conclusions

Previous study has shown that oral administration of silk peptide to mice stimulates NK cell proliferation and maturation, and activates NK cell cytotoxicity (Jang et al. 2018). In this study, we demonstrated that silk peptide treatment enhanced NK cell activity in an NK cell line *in vitro*. Silk peptide treatment significantly enhanced the cytolytic activity of NK cells in splenocytes, enhanced NK cell maturation, and upregulated the expression of various NK cell activation receptors. Collectively, these results suggest that silk peptide stimulates NK cells, thereby influencing systemic immune functions and improving natural immunity. Thus, silk peptide could be useful as a complementary therapy to promote optimal NK cell activity in cancer patients.
